# Adjuvant imatinib treatment improves recurrence-free survival in patients with high-risk gastrointestinal stromal tumours (GIST)

**DOI:** 10.1038/sj.bjc.6603797

**Published:** 2007-05-29

**Authors:** B Nilsson, K Sjölund, L-G Kindblom, J M Meis-Kindblom, P Bümming, O Nilsson, J Andersson, H Ahlman

**Affiliations:** 1 Lundberg Laboratory for Cancer Research, Departments of Surgery at the Sahlgrenska Academy, Göteborg University, Göteborg 41345, Sweden; 2 Department of Pathology at the Sahlgrenska Academy, Göteborg University, Göteborg 41345, Sweden; 3 Department of Musculoskeletal Pathology, Royal Orthopaedic Hospital NHS Foundation Trust, Robert Aitken Institute of Clinical Research, University of Birmingham Medical School, Birmingham B15 2TT, UK

**Keywords:** adjuvant treatment, gastrointestinal stromal tumour (GIST), imatinib, *KIT*, mutation, *PDGFRA*

## Abstract

Palliative imatinib treatment has dramatically improved survival in patients with malignant gastrointestinal stromal tumours, particularly in patients with tumours harbouring activating *KIT* mutations. To evaluate the effectiveness of adjuvant imatinib after radical surgery, a consecutive series of patients with high-risk tumours (*n*=23) was compared with historic controls (*n*=48) who were treated with surgery alone. The mean follow-up period was over 3 years in both groups. Only 1 out of 23 patients (4%) in the adjuvant treatment group developed recurrent disease compared to 32 out of 48 patients (67%) in the control group. This preliminary study indicates that 1 year of adjuvant treatment with imatinib dramatically improves recurrence-free survival. Confirmation of these findings awaits the results of ongoing randomised studies.

The survival of patients with metastatic and inoperable malignant gastrointestinal stromal tumours (GIST), particularly those whose tumours have *KIT* exon 11 mutations, has improved dramatically since the introduction of imatinib mesylate into treatment protocols (Verweij *et al*, 2004). The role of adjuvant imatinib treatment in GIST, however, is unclear and is currently being investigated in ongoing trials. Four such studies, including patients have undergone radical (R0) surgery, are currently being conducted with different inclusion criteria in terms of malignant potential according to the consensus grading system of Fletcher *et al* (2002). ACOSOG Z9000 addresses treatment with imatinib (400 mg day^−1^ p.o.(orally)) for 1 year in patients with high-risk GIST with no control arm, while ACOSOG Z9001 compares imatinib treatment with placebo in patients with tumours ⩾3 cm (low, intermediate, and high-risk tumours). EORTC 62024 is designed for patients with intermediate- and high-risk GIST treated with imatinib (400 mg day^−1^ p.o.(orally)) *vs* placebo for 2 years. Finally, SSG XVIII includes high-risk GIST treated with imatinib (400 mg day^−1^ p.o.(orally)) for either 1 or 3 years.

The purpose of this study was to report our experience with adjuvant imatinib, while awaiting the results of ongoing multicentre trials. Our study consists of a single-centre, consecutive pilot series of 23 patients with high-risk GIST who have been treated with adjuvant imatinib (400 mg day^−1^) for 1 year after R0 resection. These cases are compared with historical controls from a previous population-based series (Nilsson *et al*, 2005; Bumming *et al*, 2006) with matched risk scores with respect to tumour size and maximal proliferative activity with Ki67 antibodies.

## MATERIALS AND METHODS

The pilot adjuvant imatinib study group consisted of 23 consecutive patients (11 women and 12 men; mean age 56 years, range 21–82 years) with high-risk GIST diagnosed between February 2001 and June 2005. The mean tumour size was 9.4 cm (s.d.=7.7, range 2–35 cm), and the mean Ki67 max% (maximum percentage of cells positive with Ki67 immunostains) was 7.0 (s.d. 5.0, range 2–10%). These patients received adjuvant imatinib (400 mg day^−1^ p.o.(orally)) for 12 months after R0 resection. The mean follow-up after onset of imatinib treatment was 40 months (s.d.=14, range 18–62 months). Mutational analyses of *KIT* exons 9, 11, 13, and 17 and *PDGFRA* exons 12 and 18 were performed with dHPLC and bidirectional direct sequencing in both patient groups (Andersson *et al*, 2006).

The majority of patients (19 out of 23, 83%) receiving adjuvant imatinib had tumours with mutations in *KIT* or *PDGFRA*. Seventeen patients had tumours with *KIT* exon 11 mutations (eight deletions, five missense mutations, and four duplications). One patient had a GIST with duplication in *KIT* exon 9 and one patient's tumour had deletion in *PDGFRA* exon 18. Four patients had tumours that lacked *KIT* and *PDGFRA* mutations.

There were 48 matched (with regard to tumour size and Ki67 max %) historical controls of high-risk GIST with R0 resections, including 25 women and 23 men with a mean age of 67 years (s.d. 13, range 25–87 years). Mean tumour size was 12.3 cm (s.d.=7, range 3.5–33 cm), and mean Ki67 max% was 11.7 (s.d.=11.8, range 0.5–40%) These patients had a mean follow-up of 36 months (s.d.=41, range 2–151 months). Twenty-nine patients had tumours with *KIT* exon 11 mutations (20 deletions, six missense mutations, three duplications), one tumour had a missense mutation in *PDGFRA* exon 12 and 18 patients had tumours that were wild type (WT) in *KIT* and *PDGFRA* (Table 1).

### Statistics

Continuous data from the different groups were compared using the non-parametric Mann–Whitney test. Categorical data from different groups were compared using Fisher's exact test. Recurrence-free survival (i.e. proportion of patients alive and without recurrent disease) was recorded from the time of initial diagnosis to the time of first recurrence or tumour-related death. Disease-free survival was calculated using the Kaplan–Meier method. Differences between groups were compared by the log–rank test. All statistical tests were two-sided*. P*<0.050 was considered statistically significant.

## RESULTS AND DISCUSSION

Only high-risk GIST were included in this pilot adjuvant imatinib study, in contrast to ongoing adjuvant trials that also include intermediate-risk and even low-risk GIST. The selection of patients with high-risk GIST was based on follow-up data from our previously published population-based study of 233 GIST patients (Nilsson *et al*, 2005). Given the high prognostic impact of both larger tumour size and higher proliferative index (Ki67 immunolabelling), we selected a historical control group that matched the adjuvant treatment group with respect to these parameters.

**Figure 1:**
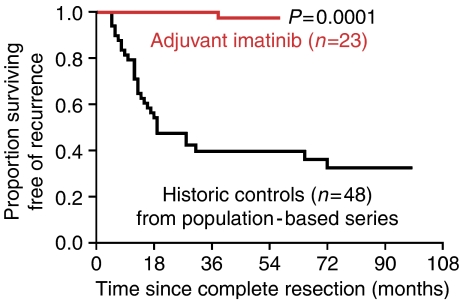
Kaplan–Meier estimates of recurrence-free survival in patients with high-risk GIST. Historical controls (black) were only treated with R0 resection (mean follow-up 36 months, range 2–151 months), while the consecutive series of patients (mean follow-up 40 months, range 18–62 months) had adjuvant imatinib (400 mg day^−1^) for 1 year after radical surgery (red). In the latter group only one patient has developed recurrence.

The estimated recurrence-free survivals for the adjuvant treatment and control group are presented in Figure 1. Only 1 out of 23 (4%) patients developed recurrent disease in the treatment group in contrast to 32 out of 48 (67%) in the control group. In the current series, as well as in our large population-based series (Nilsson *et al*, 2005), the vast majority of recurrences in high-risk GIST are within 2 years of diagnosis. Notably, there was no recurrent disease in the treatment group during the first 2 years after diagnosis.

The only recurrence in the treatment group was in a 10-year-old girl with a small intestinal GIST; after five resections for recurrences over a 12-year period (the last being a hemi-hepatectomy for liver metastasis), she received adjuvant imatinib for 12 months. Twenty-two months after termination of imatinib treatment, a small lung metastasis was detected and removed. There is a distinct, rare subgroup of malignant GIST associated with a protracted clinical course despite multiple distant metastases over a long period of time. These have a distinct predilection for young females and have been shown to lack *KIT* and *PDGFRA* mutations (Persson *et al*, 1992; Miettinen *et al*, 2005). Interestingly, this girl's GIST also lacked *KIT* and *PDGFRA* mutations. These findings are consistent with previous reports that GIST with *KIT* exon 11 mutations have the highest response rate to palliative treatment with imatinib.

We have recently shown that *KIT* mutation status is an independent prognostic factor in GIST in addition to tumour size and proliferative index (Andersson *et al*, 2006). The overall mutation frequency was somewhat higher in the adjuvant treatment group (19 out of 23, 83%) compared to the control group (30 out of 48, 63%). The reasons for this are probably technical (older paraffin-embedded material in the control series and some analyses performed on fresh frozen material in the adjuvant treatment group) (Andersson *et al*, 2006; Kindblom *et al*, 2006). However, the frequency of *KIT* exon 11 deletions, which have been found to be associated with a more aggressive clinical course (Andersson *et al*, 2006), was similar in the two groups, with 8 out of 17 *KIT* mutations in the adjuvant treatment group and 20 out of 29 *KIT* mutations in the control group were deletions.

In summary, our study shows that 1 year of imatinib (400 mg day^−1^ p.o.(orally)) treatment after R0 resections for high-grade GIST dramatically reduces the risk of recurrent disease. These results await confirmation with ongoing prospective adjuvant treatment trials.

## Figures and Tables

**Table 1:** Clinical data and tumour characteristics

					**Receptor tyrosine kinase mutations**					
**Group**	**Number of patients**	**Mean tumour size (cm)**	**Ki67 max%**	**Mitotic rate (/50 hpf)**	***KIT ex 11***	***KIT ex 9***	***PDGFRA ex 18***	***PDGFRA ex 12***	***WT***	**Mean follow-up (months)**
Adjuvant imatinib	23	9.4	7.0	6.2	del: 8	dupl: 1	del: 1	0	4	40
	F: 11	s.d. 7.7	s.d. 5.0	s.d. 2.6	miss: 5					s.d. 14
	M: 12	range 2–35	range 2–10	range 2–10	dupl: 4					range 18–62
										
Historic controls	48	12.3	11.7	6.8	del: 20	0	0	miss: 1	18	36
	F: 25	s.d. 7	s.d. 11.8	s.d. 3.3	miss: 6					s.d. 41
	M: 23	range 3.5–33	range 0.5–40	range 2–10	dupl: 3					range 2–151

Abbreviations: del=deletion; dupl=duplication; ex=exon; F=female; hpf=high power fields; M=male; miss=missense mutation; PDGFRA=platelet-derived growth factor receptor *α*; WT=wild type (in *KIT* and *PDGFRA*).
